# The gender dimensions of social networks and help-seeking behaviors of young adults in Soweto, South Africa

**DOI:** 10.3402/gha.v9.31138

**Published:** 2016-06-03

**Authors:** Kathryn Meagley, Brittany Schriver, Rebecca S. Geary, Rebecca Fielding-Miller, Aryeh D. Stein, Kristin L. Dunkle, Shane A. Norris

**Affiliations:** 1 Department of Behavioral Sciences and Health Education, Rollins School of Public Health, Emory University, Atlanta, GA, USA; 2 MRC/Wits Developmental Pathways for Health Research Unit, Department of Paediatrics, University of the Witwatersrand, Johannesburg, South Africa; 3 Department of Global Health, Rollins School of Public Health, Emory University, Atlanta, GA, USA; 4 Department of Population Studies, Faculty of Epidemiology and Population Health, London School of Hygiene and Tropical Medicine, London, UK; 5 Gender and Health Research Unit, South African Medical Research Council, Pretoria, South Africa

**Keywords:** health services, help-seeking behavior, social support, reproductive health, sexual health, young adult, South Africa

## Abstract

**Background:**

Young people constitute a major proportion of the general population and are influenced by a variety of factors, especially in regards to seeking help. An understanding of help-seeking behaviors among young people is important for designing and implementing effective targeted health services.

**Methods:**

We conducted in-depth interviews with 23 young adults aged 21–22 years in Soweto, South Africa, to explore the gender dimensions of social networks and help-seeking behaviors.

**Results:**

We found that young men had larger peer social networks than young women and that young women's social networks centered on their households. For general health, both young men and young women often sought help from an older, maternal figure. However, for sexual health, young men consulted their group of peers, whereas young women were more likely to seek information from one individual, such as an older female friend or family member.

**Conclusion:**

These differences in help-seeking behaviors have important implications for the delivery of health information in South Africa and how health promotion is packaged to young men and women, especially for sexual and reproductive health issues. Peer educators might be very effective at conveying health messages for young men, whereas women might respond better to health information presented in a more confidential setting either through community health workers or mHealth technologies. Provision of or linkage to health services that is consistent with young people's health-seeking behavior, such as using peer educators and community health care workers, may increase the reach and utilization of these services among young people.

## Introduction

Young people increasingly constitute a major segment of the population, particularly in low- and middle-income countries. In response to the growing adolescent health burden, international organizations have promoted the development and scale-up of health services that are ‘youth-friendly’ (1–5). The Youth Friendly Services (YFS) program in South Africa is one such example of a local program that has been scaled-up to a national level. This program, implemented in primary health care facilities, aims to improve the sexual and reproductive health of young men and women (6). However, there is evidence that the implementation and impact of the YFS program in Soweto is limited (7).

Understanding the factors that drive how and when a young person seeks help, from informally consulting personal social networks to attending formal health care services, is important for effective health promotion and service delivery (8–13). Previous research has found that social networks and relations influence youths’ help-seeking behavior related to mental health, substance abuse, and sexually transmitted infections (STIs) (14–17). These networks and behaviors are highly influenced by sociocultural factors, such as gender (8, 9, 17–20). There is a greater need to understand the intersection of the factors that drive help-seeking behavior processes in young people, especially in low- or middle-income countries, and how that may impact utilization of health services. Alongside efforts to improve implementation of YFS, a deeper understanding of the gender dimensions of young people's help-seeking behaviors could inform strategies to promote available health services and link young people to care.

This study aims to investigate the gender differentials of help-seeking behaviors and the role of community and social support network relationships in young men and women's help-seeking behavior in urban Soweto, South Africa.

## Methods

### Setting and sampling

The Birth to Twenty (BT20) cohort is the largest and longest-running study of child and adolescent health and development on the African continent and has been described in more detail elsewhere (21). A subset of 50 BT20 cohort participants from Soweto, South Africa, an urban township of approximately 1.3 million individuals situated to the southwest of Johannesburg, was randomly selected from the full cohort to pilot the periodic Young Adolescent Health Survey (22). We purposively sampled 23 black African young men and women for participation in this study based on gender (14 female/9 male) and utilization of health services within the last 6 months (15 users/8 non-users).

Both the Emory University Institutional Review Board (ID 58317) and the Human Ethics Research Committee of the University of Witwatersrand (ID M120138) approved this study. Informed written consent was obtained from all participants.

### In-depth interviews

We developed an in-depth interview guide that covered social networks, definitions of health, and perceptions of health services. Four pilot interviews with fieldworkers were conducted to assess the cultural appropriateness and relevance of the questions. We then conducted five pilot interviews with members of the BT20 cohort to determine and refine the interview method. Three interview methods were piloted: 1) a semi-structured one-on-one interview in English, 2) a semi-structured one-on-one interview in a native language (e.g. Zulu), and 3) an interview in English with a native language research assistant present for translation if the participant felt more comfortable using his or her native language during the interview. These pilot interviews indicated that participants were most comfortable expressing their opinions in the semi-structured one-on-one in-depth interviews between an investigator and the participant in English. The final interview guide was found to be sufficient for the purposes of the study, culturally appropriate, and understood by the participants.

As part of the interview, we asked participants to draw a relationship map illustrating the people close to them. These relationship maps were used to facilitate a richer discussion of their important relationships and social context.

Project staff invited participants to be interviewed by phone. We held interviews in private interview rooms at the BT20 cohort offices. All interviews were digitally recorded.

### Data analysis

We conducted all interviews in English as per the pilot results and because English literacy in Soweto is high. Interviews varied in length between 45 minutes and 2 hours. All interviews were transcribed verbatim. We used modified grounded theory to guide our work (as described by Borgatti (23) and Strauss and Corbin (24)). A literature review informed the development of our data collection tools. Informed by the themes of availability, accessibility, and acceptability as previously discussed in the literature, we developed initial codes deductively (25–28). We allowed additional themes to emerge inductively throughout the data collection and analysis.

Two investigators (KM and BS) read the transcripts independently and separately developed codebooks based on themes from the literature review and core themes that emerged through the interviews. This iterative process allowed us to ensure the codes were grounded in and produced by the data itself while in line with accepted theories of help-seeking behavior. The two codebooks were then synthesized and consolidated and both investigators agreed upon the code definitions.

Inter-coder reliability was examined for three interviews to ensure consistency in coding and interpretation. Discrepancies in coding were discussed and a consensus was reached. Prior to analysis, we merged the independently coded transcripts so that the final transcripts used for analysis included both of the independently coded text. The relationship maps were scanned and coded based on the corresponding themes identified in the interview. The analysis was conducted using MAXQDA 10 qualitative analysis software (29).

## Results

The results are grouped into two thematic areas: 1) social support and 2) help-seeking behavior.

### Social support

The daily lives of these young adults were shaped by familial, educational, and financial responsibilities; they provided and cared for their family while balancing domestic obligations and personal relationships. In general, young men talked more about having large social networks of close relationships than young women, whereas young women spoke more about having to make a greater effort to maintain relationships with those who lived outside of their household.

#### Family structure and father absence

All of the participants were living with family members and none were married. Five individuals lived with their married biological parents, nine participants lived in single-parent households, and seven participants lived in households headed by their grandmother. Two young men lived with a relative other than an immediate family member or grandparent.

There was a general lack of older male roles models within social and familial networks, which had significant implications for male participants in particular. A 22-year old male discussed the personal consequences of being raised in a single-parent household:

I never had a role model in my life. Like … if I had a figure, someone I can say I look up to … I can't talk to anyone. Like if I had a problem with a woman … traditionally there are things that I don't talk to my mother to. (22-year-old male, raised in a single-parent household)

Women also found the lack of male participation in familial networks difficult. Of the 14 female participants, one was pregnant and six had children, all of whom were under 5 years old. Young women who had children talked about spending a great deal of time on their domestic responsibilities and noted that they and their families were wholly responsible for raising their children. None discussed sharing these responsibilities and experiences with the biological fathers of their children. This lack of involvement of the biological father put financial stress on the women. One woman spoke about the pressures of financially supporting her family and taking care of her child:

After getting a baby … [my family] did nothing. They didn't help. I even failed my [high school exams]. Like, there was nothing, I had to stay and take care of the baby. […] I think my son was about 2 months, I got a job. I was making about 1,400. And the transport was 900. I was going to be left with 500. But my grandmother wanted me to buy food at home. I had to give her 300 and left with only 200. (21-year-old female, employed; 4-year-old child)

Much of the young women's emotional support came from individuals in their household, usually from their mother or grandmother. When they had a general health problem or other concerns (for example regarding finances), they talked about consulting a family member. However, if their problems concerned sexual relationships, they usually sought support from an older sibling.

#### Peer groups

Participants did not talk about forming any new close friendships, but rather spoke about long-standing relationships. There was a difference in peer group size between the young women and men.

Most of the women interviewed had a social circle that included between three and eight meaningful relationships. However, within these groups, most of the young women identified one or two close friends with whom they could consult if they had a question or problem. Young women were very deliberate about selecting with whom they would share their problems. One young woman expressed this concern about her peers gossiping:

OK, I don't have friends that way. Just people I know and socialize together with at school. […] Girls talk a lot about you. They gossip. Too much drama. That's why I talk to my sister a lot. All of it. (21-year-old female, no children)

The fear of gossip was a common theme among young women. As a result, young women were more likely to keep the problems to themselves or to consult a family member.

In contrast, men spent more time with their group of friends than women. Many of the young men talked about having peer networks that they would see and consult with on a daily basis. When asked what they talked about, many male participants discussed how they shared their issues, but debated the merits of the information they had obtained previously. For example, one man recalled how when he needed advice he sought guidance from his peers:

I'm chilling with the friends, I'm asking just so so … like, me and my friends […] we get advice from someone and bring those things to the corner and talk about it and come back. You can just give advice just there at the corner. (21-year-old male, living in a single-parent household)

Romantic relationships were one of the few close relationships that young women had outside of their family. Women viewed romantic relationships as a source of emotional and financial support, even for those who thought that their boyfriends had other concurrent relationships. Whereas the young women had a tendency to consult their boyfriends if they needed support, fewer young men reported consulting their girlfriends. One young man suggested that he did not consult his girlfriend because he did not see her a lot and because he saw his problems as personal issues:

When I go and I'm in these relationship[s] with [a] girlfriend, she also has a place where she stays, so sometimes it's only for a week […] But we never speak, like talk about life. It's just these short term so you can't just tell her things. I just keep it there (points to heart) 'cause with this girlfriend I know it's only fine. You never bring up that side; if you have a bad side she obviously can't be with you. That's why you leave your problems at home. (22-year-old male, student)

#### Church support

Several participants discussed how they had formed close relationships with peers in their religious institutions when they were younger and first started going to church. They remained close and would regularly spend time with these people outside of the church setting. A few young women discussed how they had been paired with older mentors within their church for guidance. However, these relationships seemed to have waned with age. Both young women and young men identified their pastors as confidants whom they could approach about problems at home, although not about romantic relationships.

#### Social media

Young women described social isolation following high school due to their domestic, employment, and educational responsibilities. They felt that they had to make a conscious effort to maintain relationships, but due to time constraints at home and work they had only limited time to catch up with long-standing friends. One female participant emphasized the importance of social media in order to maintain relationships:

I used to have a lot of friends in high school. I'm still close to some of them. But we hardly see each other now, 'cause some of them don't live here anymore, some are always in school. But when I'm at work, they are at home. But, when I'm off they're at school, so we hardly see each other between … mostly talk through the phone … or WhatsApp … or Mix It, or Facebook. (22-year-old female, employed, no children)

Only one male participant reported using social media to maintain a relationship, because his brother was incarcerated. We found that young men generally spent more time with their friends in person.

### Help-seeking behavior

Both young men and women went to the public health clinics for general illnesses, reproductive health issues, contraceptive services, and HIV/STI testing. Young women sought health care services for themselves as well as for others, whereas men sought care for themselves only. When women went to clinics for themselves, it was mostly for contraceptive services or reproductive health issues. Young women took their young children to get vaccinated and helped their grandmothers go for annual check-ups. Although men generally attended the clinics only for acute general illness, they sometimes also went for medical male circumcision or tuberculosis treatment. None of the participants reported seeking health care from traditional healers.

#### General health and illness

When the participants had a question about their general health, both young men and young women preferred to consult an older maternal figure who was close to them and who they considered to be knowledgeable, such as their mother, grandmother, aunt, or neighbor. Some participants reported that they had learned home remedies for common sickness from their mothers. Both young men and young women explained they went to these people because they were the ones who raised them and therefore knew them best. These two excerpts demonstrate how both young men and women valued their mother's knowledge when they were feeling sick:

I would say she [was] the one who carried me until … until I was two years old. So … she knows what's best for me. What can I take … she knows how I look. She knows when I'm sick. So I go to my mom. (22-year-old male, employed, living with older relatives including mother)

I would talk to my mother […] Because she's the older one and she's been through some things that I have [gone] through so it's going to be much easier to ask her about such things. (22-year-old female, student, living with older female relatives)

If an individual did not have the answers or the symptoms persisted, he or she would either seek out another maternal figure close to them (if such a person existed) or would go to professional health care services, either at local or private clinics.

### Sexual and reproductive health

Both men and women would collect information about sexual and reproductive health concerns before making a decision based on severity to attend the clinic. Unlike general health information and illness, participants’ sociocultural context affected help-seeking behaviors for sexual and reproductive health in very specific ways. Both young men and young women found it embarrassing and uncomfortable discussing their sex lives with their parents, grandparents, or religious mentors. One young woman described her reluctance to consult her pastor:

I'll go to my friend, not my lady pastor. Yeah, 'cause she'll open the Bible, quoting verses, you know. It's a sin, dating and all that. It's too much. (21-year-old female, no children)

Young women were consistently cautious with whom they consulted about reproductive and sexual issues, once again citing fear of gossip among their peers. Young women chose rather to consult a close female friend, a boyfriend, or the Internet.

If young women preferred to go to one person, they generally chose a slightly older female, usually a close friend, sibling, or cousin not more than 10 years their senior. Because of a lack of trust in sexual health information received from their peers, women were more likely than men to corroborate information through additional sources, such as the Internet. After collecting information, women would make a decision to seek additional medical attention based on the information gathered. For example:

Even though [my female cousin] may be … maybe 2 years [older]. Because it's easier to talk to her, I guess, like talk to her freely. Even though she's not going to give me that right answers that I need. […] Honestly, first I start going to the Internet. Then I will check, check, check, check. Then … if it's serious, if that thing is serious then I have no choice, I have to go to the clinic. (22-year-old female, student)

Young women also went to their boyfriends with concerns about their sexual health. Two young women described a past episode of an acute sexual health problem. Rather than going to their female counterparts, they chose to go to their boyfriend first. A few of the young women said they went to their boyfriends if they had a problem because they trusted that their boyfriends would not gossip. Their boyfriend either escorted them to the clinic or provided them with money to attend a private doctor. One young woman explained her reasoning:

My friend I don't talk to, I don't talk to her that much. 'Cause friends like, they like to gossip. You go and tell her something, then they take it to the other person. 'Cause my boyfriend is a guy. Guys don't do those things. (21-year-old female, employed)

Similarly to young women, young men also discussed seeking advice about condoms and sex from a slightly older male, who they could relate to on a personal level, but who was more experienced and therefore believed to have more accurate information; to illustrate, one young man explained how he consults an older friend:

He's older than me, possibly about 9 years. So, he's been through these things. He knows, basically what's going on. And I will go to him and tell him, OK my friend, [I'm] in [a] situation like this … (21-year-old male, lives with both parents)

Many young men said that they wanted multiple perspectives before making a decision. Because information was exchanged within a group setting, they strategically acquired different perspectives and opinions without having to actively seek out information about sexual and reproductive health from formal health care settings. For young men, their male peers and ideas of masculinity primarily influenced their health behaviors. One male participant recounted how he felt pressure from his circumcised friends to have the procedure:

Now only two are circumcised, and two are uncircumcised. I went to the clinic today and made a booking. You know it's like, you're not [a] real man. You're [a] man, but you're not man enough. (21-year-old male, lives with both parents)

Although family members encouraged men to attend health care services when issues arose, men's behaviors were more likely to be influenced by their male peers.

## Discussion

It has previously been shown that a young person's decision to seek help and health care services related to mental health, substance abuse, and STIs is heavily influenced by their social networks (14–17). However, we found that the support networks that were used in regards to help-seeking behaviors differed between genders. Many of the social networks for women revolved around their households, while men's centered more on their peers. For young women, the combination of domestic obligations with financial and social independence had the effect of constricting their social network to their immediate household and family. Fear of gossip regarding personal and health issues perpetuated this social isolation. Most women relied heavily on a select few close relationships and older female figures within their networks.

Male participants described a general lack of older male role models within their social network and therefore often turned to their peers for information instead – as has been observed in other South African studies (30, 31). Men generally had fewer time constraints and financial and domestic obligations, so they had more time to spend with their peers outside of their household. As a result, male participants had larger social networks than did women and did not feel the need to use technology and social media in order to maintain them.

Help-seeking behavior also differed based on the type of service that was sought. For example, young men and women both consulted a family matriarch first for questions about general health, but found other routes of information for questions regarding reproductive and sexual health. These alternative routes for sexual health information fell along gender lines. Women chose to consult a slightly older female friend or family member, the Internet, or their boyfriend. Men chose to consult an older male relative or a larger peer group. Both men and women acknowledged that not all information from heir peers was sound. Women would corroborate this information through more private conduits such as the Internet, whereas men would consult other peers for consensus. Peer-related social desirability influencing behavior and decision making in adolescent young men has been noted in other studies as well (8, 32).

Although many factors influenced the paths taken to seek out help, when participants sought formal health care, they typically went to a public health clinic or a private doctor. As young people seem willing to utilize both public and private health care services in this urban setting, it is important that these services be high quality and age- and gender-appropriate. It is also important to consider how services can address the pathways men and women take to seek help to better target interventions and service promotion. For example, because of their reliance on social networks, peer educators have been found to be particularly effective in encouraging young men to seek out reproductive health services (33, 34). Meanwhile, community health workers or mHealth programs may be more appropriate for reaching women who have domestic responsibilities and greater concerns with confidentiality (35–37). Understanding and utilizing different pathways offers a greater opportunity to reach youth effectively and provide them with necessary health education and services.

## Limitations

Gender is a complex, multifaceted construct that intersects with other key determinants such as socioeconomic status, race, religion, class, culture, and time. As such, this investigation has several limitations. All interviews were made by Caucasian women – the gender and race imbalance with interviewees could have resulted in a bias in response. Additionally, all respondents were of similar SES, race, and culture. Given the study population, the scope of this paper was limited to investigating the gender dimensions of help-seeking behavior without a greater discussion of intersectionality of other key determinants. Although this investigation offers a more thorough understanding of the process of help-seeking behaviors among youth in urban South Africa, more work will need to be done to generalize these findings to other contexts, both in rural South Africa and on a more global level.

## Conclusions

In South Africa, gender-differentiated social networks and obligations shape the pathways of help-seeking behaviors. This has implications for how to best disseminate and communicate important health information. However, in order to improve health-seeking behaviors, it is important to understand the pathways of help-seeking behaviors and sources of information. Health promotion campaigns and the distribution of health information need to be culturally sensitive to the social and gender structure in order to facilitate a higher sense of individual agency, so that youth will seek appropriate health services.

In the instance of YFS in South Africa, it may be necessary to consider how young people seek help in promoting health services and expanding service beyond health care facilities. Peer educators, community health workers, and mHealth programs may be beneficial as approaches, but may impact help-seeking behavior differently for men and women. Different genders may require different approaches, so a constellation of services and approaches is recommended to ensure health service access and utilization among all young people.

## Paper Context

At 1.8 million, the current cohort of young people is the largest in history so far. The adolescent health agenda is gathering pace, and South Africa has a program of youthfriendly health services. However, few studies have examined the gender dimension of social networks and help-seeking behaviors among youth. In South Africa, gender-differentiated social networks and obligations shape pathways of help-seeking behaviors. This can inform the design and implementation of health services, potentially increasing their reach and utilization.
